# A high sensitivity acetone gas sensor based on polyaniline–hydroxypropyl methylcellulose core–shell-shaped nanoparticles

**DOI:** 10.1039/d2na00647b

**Published:** 2022-11-15

**Authors:** Ji-Sun Kim, Jun-Ho Byeon, Sungmin Kang, Jin-Yeol Kim

**Affiliations:** School of Advanced Materials Engineering, Kookmin University Seoul 136-702 Korea jinyeol@kookmin.ac.kr +82-2-910-4663; Advanced Technology Research Department, LG Japan Lab Inc. 413-14, Higashi Shinagawa, Shinagawa-ku Tokyo 140-0002 Japan; Institute of Innovative Research Laboratory for Future Interdisciplinary Research, Tokyo Institute of Technology Yokohama 226-8503 Japan

## Abstract

Core–shell-shaped nanoparticles (CSS-NPs) with polyaniline emeraldine salts (PANi) in the core and hydroxypropyl methylcellulose (HPMC) and heptadecafluorooctanesulfonic acid (C8F) shells, *i.e.*, C8F-doped PANi@HPMC CSS-NPs, were synthesized as a gaseous acetone sensing material with high sensitivity and humidity stability. The HPMC was chemically combined on the positively charged PANi NPs' outer surface, allowing it to efficiently detect acetone gas at concentrations as low as 50 ppb at 25 °C. To impart humidity stability, C8F was employed as a hydrophobic dopant, and a valid signal could be reliably detected even in the range of 0–80% relative humidity. The sensing material's structural analysis was conducted using scanning electron microscopy, transmission electron microscopy, energy-dispersive X-ray spectroscopy, and infrared spectroscopy, and in particular, the reaction mechanism with acetone gas was detected through a spectroscopic method. Thus, these findings illustrate the potential as a novel sensing material to detect acetone gas at a trace level of less than 1 ppm in human respiratory gas.

## Abstract 2

Core–shell-shaped C8F-doped PANi@HPMC CSS-NPs were synthesized as sensing materials. The sensor can effectively detect less than 50 ppb of acetone gas at room temperature, especially with relatively high selectivity.

## Graphical abstract

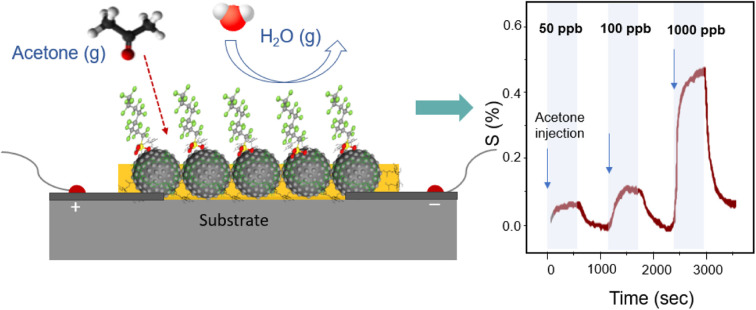

## Introduction

π-Conjugated conductive polymers (CCPs), including polypyrrole (PPy)^1–3^ and polyaniline (PANi),^4^ which respond to their environment or external stimuli to produce a dynamic and reversible change in properties, have recently attracted considerable attention due to their potential applications in optoelectronic devices and electrochemical sensors.^5–9^ Recently, electrochemical sensors based on CCPs^10–16^ have emerged as very promising materials for gas sensors due to changes in their resistance in the presence of reducing or oxidizing harmful gases. Charged carriers are created in the π-conjugated chain by chemical doping, their movement affects the conductivity, and the resistance is changed by the adsorption of gaseous species in the electrical resistance of CCP, as an electrical conductor. When a positively charged doped CCP-based sensor is exposed to an oxidizing gas, its resistance decreases in proportion to the adsorbed gas concentration. However, when reducing gas components including ammonia is adsorbed, their resistance values increase. However, CCPs become redox-active because they alter the addition or removal of electrons from the conjugated chain backbone of CCPs by doping or de-doping with dopants or oxidizing/reducing gas components. These CCP-based sensors also offer several appealing characteristics that have been widely proposed for gas detection reported so far.^17,18^ First, they operate at ambient temperature, and the sensing materials can be designed to detect certain compounds with minimal cross-sensitivity to other compounds. However, to achieve the sensor's performance required for practical usage, higher sensitivity is needed, and the development of a sensing material with efficient response characteristics even under humidity conditions at room temperature remains an essential effort.

To address the above problems, we developed core–shell-shaped nanoparticles (CSS-NPs) comprising PANi in the core and hydroxypropyl methylcellulose (HPMC) and heptadecafluorooctane sulfonic acid (C8F) shells, *i.e.*, C8F-doped PANi@HPMC CSS-NPs, specifically employed as an acetone gas sensing material. PANi is a p-type semiconductor with selectivity to specific organic components that have the structure of emeraldine salts. Structurally, it is a positively charged conductive polymer particle with a spherical surface surrounded by HPMC molecules that are sensitive to acetone gas. Particularly, because of its hydrophobic properties, C8F is placed on the surface of PANi nanoparticles and not only influences the electrical resistance value but also functions as a dopant to minimize the resistance change due to humidity. It has the benefits of high sensitivity, moisture stability, and low-temperature detection.^11–15,19^ Recently, Zhang *et al.*^15^ reported an acetone sensor made of a ZnO/S–N graphite QD/PANi composite with an enhanced response of the 500 ppb level at 25 °C.

**Fig. 1:**
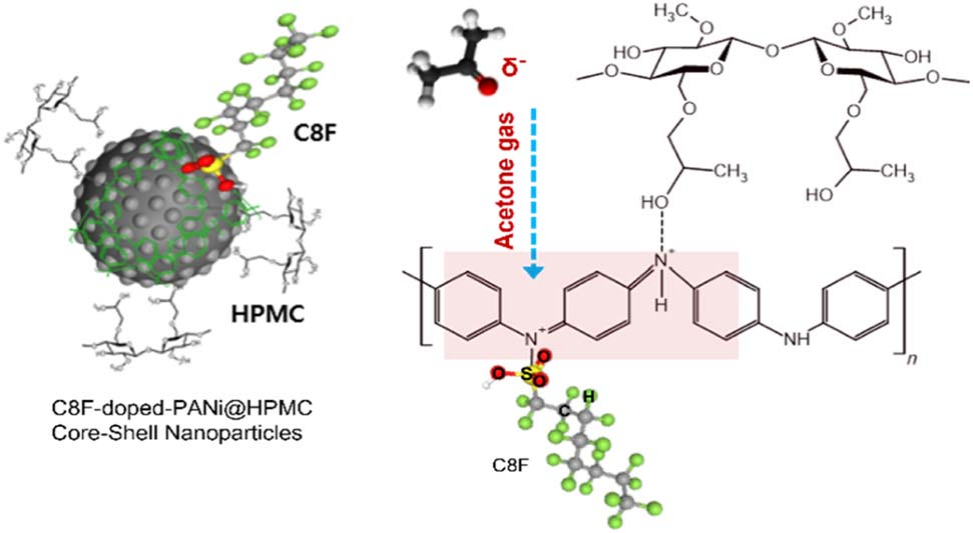
Schematic illustration and chemical structure of core–shell-shaped nanoparticles (CSS-NPs) composed of a heptadecafluoro-octanesulfonic acid (C8F)-doped polyaniline emeraldine salt (PANi ES) core and hydroxypropyl methylcellulose (HPMC) shell in the nanocomposites as an acetone sensing material.

In this research, we developed C8F-doped PANi@HPMC CSS-NPs using micelle templating in an emulsion polymerization approach. HPMC was employed as a polymer surfactant to generate spherical micelles; some of them were connected to the PANI's NH group backbone through electrostatic interaction during polymerization and finally formed as a capping layer on the spherical PANI's surface. Because of its hydrophobic properties, C8F is placed on the surface of conductive PANi NPs and functions as a dopant to not only enhance electrical conductivity but also minimize resistance changes due to moisture. Furthermore, Fig. 1 depicts the schematic and chemical structures of CSS-NPs made of C8F-doped PANi capped with HPMC. Thus, the C8F-doped PANi@HPMC CSS-NP sensor in this research efficiently detected acetone gas at concentrations as low as 50 ppb even at ambient temperature and could detect a consistent signal at a relative humidity (RH) ranging from 0–80%. It is thought that a human respiratory gas test could be useful for medical diagnosis.

## Results and discussion

Fig. 1 shows the C8F-doped PANi@HPMC CSS-NPs' illustration and structure for the C_3_H_6_O gas sensor. Monodispersed core–shell-shaped nanoparticles were developed using micelle polymerization with HPMC surfactant in an aqueous solution. The generated products were purified through a centrifugation approach employing methanol and deionized water solution, resulting in a high-purity monodisperse aqueous dispersion. It has a conventional core–shell structure, in which HPMC and C8F produced as a capping layer are combined on the backbone of the spherical PANi. As a p-type semiconductor, the NH^+^ functional group in the positively charged PANi polymer chain is bonded with C=O in a weak hydrogen bond by charge–charge interaction when C_3_H_6_O molecules are adsorbed. In other words, the charge density in the backbone of PANi is reduced, increasing electrical resistance. However, when C8F-doped PANi@HPMC CSS-NPs were employed as sensing materials, C_3_H_6_O acted as a reducing agent, reducing the PANi backbone's doping level. Likewise, similar investigations on the reaction mechanism between a sensor employing PANi and acetone gas have been reported in several previous research studies.^20,21^

**Fig. 2:**
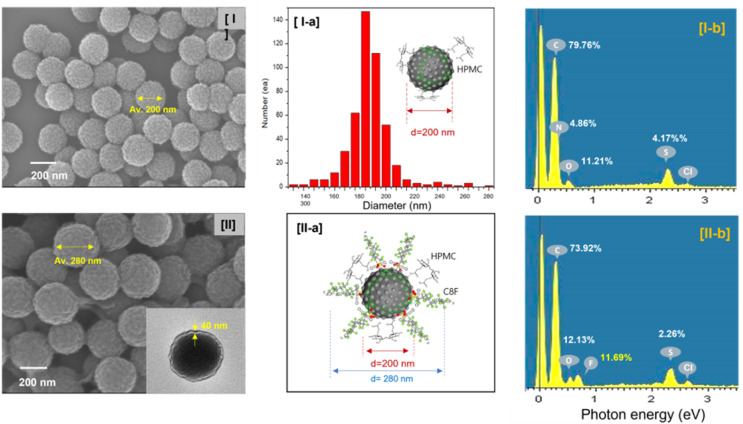
Scanning and transmission electron microscopy images of the (I) C8F-undoped PANi@HPMC CSS-NPs and (II) C8F-doped PANi@HPMC CSS-NPs, respectively, and their illustration and structures of core–shell-shaped nanoparticles. (I-a) A plot of the particle distribution diagram of C8F-undoped PANi@HPMC CSS-NPs by size. (I-b) and (II-b) EDS spectra and their elemental analysis data for each sample.

**Fig. 3:**
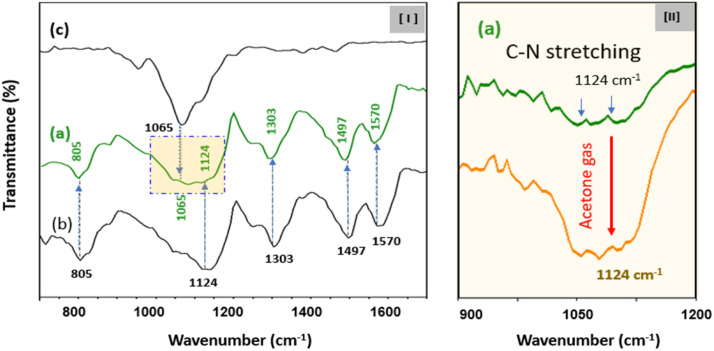
(I) Fourier transform infrared (FT-IR) transmission spectra of (a) C8F-doped PANi@HPMC CSS-NPs, (b) pure HPMC molecules, and (c) a PANi particle alone. (II) FT-IR transmission spectrum when C_3_H_6_O gas is adsorbed on C8F-doped PANi@HPMC CSS-NPs. (Intensity of the 1124 cm^−1^ peak increased significantly).

Morphological structures revealed that CSS-NPs were well produced from the scanning electron microscopy (SEM) images of PANi@HPMC CSS-NPs without C8F and C8F-doped PANi@HPMC CSS-NPs, respectively, as demonstrated in Fig. 2. Particularly, the figure placed into the SEM image of Fig. 2(II) illustrates a transmission electron microscopy (TEM) image of one specific particle. As demonstrated in the figure, CSS-NPs consist entirely of monodisperse spherical nanoparticles with average diameters of 200 and 280 nm. HPMC employed to produce micelles and C8F employed as a hydrophobic dopant were produced with a thickness of about 40 nm on the shell of PANi particles during polymerization. In the PANi particles synthesized without C8F, only a 2–3 nm thin HPMC layer exists, as demonstrated in Fig. 2(I). Here a thin C8F layer containing fluorine (F) atoms sufficiently covered the PANi nanoparticles' entire surface. Energy-dispersive X-ray spectroscopy (EDS) spectra were employed to examine the component content of nanoparticles, as demonstrated in Fig. 2(II-b). The mass percentage of fluorine atoms on the C8F-doped PANi@HPMC CSS-NPs was estimated and compared with the content of other components. The mass percentage of F atoms was found to be 11.69% for C8F-doped PANi@HPMC CSS-NPs. Obviously, in the spectrum of PANi@HPMC CSS-NPs without C8F in Fig. 2(I-b), it was seen that F atoms were not present at all. FT-IR spectroscopic analysis was performed to characterize C8F-doped PANi@HPMC CSS-NPs, as demonstrated in Fig. 3(I-a). For a comparative analysis, Fig. 3(I-b) and (I-c) also illustrate the IR transmission spectrum of pure HPMC molecules in the outer shell and PANi particles alone in the core, respectively.

On the IR spectrum of the obtained C8F-doped PANi@HPMC CSS-NPs, the peaks at 1570, 1497, 1303, 1124, and 805 cm^−1^ are attributed to the C–C stretching of a quinoid ring, C=C stretching of a benzene ring, C–N stretching of an aromatic ring, stretching of a charged C–NH^+^ group, and C–H out of plane in the 1,4-distributed benzene ring, respectively.^22,23^ The characteristic peaks seen at 1065 cm^−1^ are attributed to the C=O group present in the HPMC shell on the PANi CSS-NPs’ surface. Here, it was verified that the intensity of this 1065 cm^−1^ peak increased with increasing HPMC shell thickness.

The reaction mechanism when C_3_H_6_O gas was injected into C8F-doped PANi@HPMC CSS-NPs was explained using spectroscopic analysis. In relation, Fig. 3(II) demonstrates the observation of changes in the IR absorption peak when C_3_H_6_O gas is adsorbed onto C8F-doped PANi@HPMC CSS-NPs. As demonstrated in the figure, when a certain amount of C_3_H_6_O gas is adsorbed, it was seen that the 1124 cm^−1^ stretching band's intensity caused by the positively charged C–NH^+^ group in the PANi backbone increased substantially. These findings also tended to recover reversibly, when the C_3_H_6_O gas was withdrawn.

The NH^+^ group of the PANi backbone interacts with the C=O group of the C_3_H_6_O molecule through charge–charge coupling. Alternately, it can be thought of as a connection between the NH group of PANi and the oxygen atom of C_3_H_6_O by a weak hydrogen bond.

**Fig. 4:**
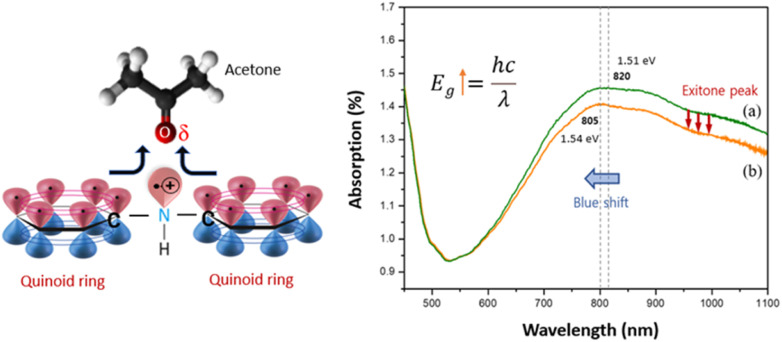
Schematic illustration of the C_3_H_6_O gas sensing mechanism and UV/vis absorption spectrum of the C8F-doped PANi@HPMC CSS-NP sensing material and the spectrum when exposed to C_3_H_6_O gas, respectively; (a) before exposure to C_3_H_6_O gas and (b) after exposure to C_3_H_6_O gas. When the oxygen atom of C_3_H_6_O is exposed to positively charged excitons distributed in the p-orbital of the quinoid ring of the PANi chain, some charged carriers move towards the oxygen atom of the C_3_H_6_O molecule. As a result, the number of excitons in the quinoid ring of the PANi chain is relatively reduced, and their band gap (*E*_g_) and electrical resistance (Ω cm) change.

Fig. 4 illustrates the C8F-doped PANi@HPMC CSS-NP's UV/vis absorption spectrum and the spectrum when exposed to C_3_H_6_O gas, respectively. As demonstrated in the figure, an absorption peak attributed to the positively charged excitons contained in the PANi chain was detected at 820 nm. At this time, the bandgap energy associated with the π–π* transition was computed to be 1.51 eV. However, these absorption wavelengths moved to 805 nm (bandgap energy: 1.54 eV) with exposure to C_3_H_6_O gas, and the absorption intensity also substantially decreased. This is because acetone gas is chemically bound to the PANi molecule by charge–charge interaction, and some of the positively charged excitons of the positively charged PANI move toward the oxygen atom of the acetone molecule, reducing the number of excitons in the PANi chain. Thus, the PANi chain's electrical resistance (Ω cm) tends to increase. The PANi chain's band gap energy (*E*_g_) also increased as the amount of acetone increased.

**Fig. 5:**
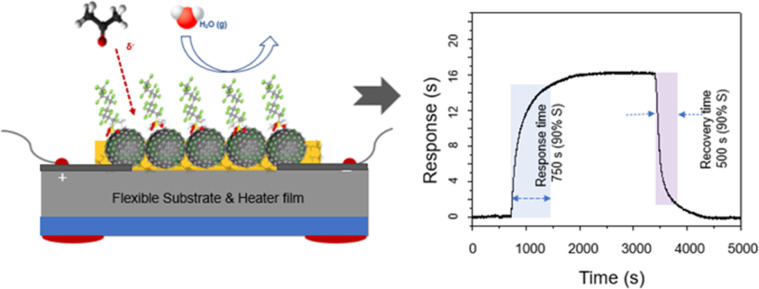
Schematic of the sensor array employing C8F-doped-PANi@HPMC CSS-NPs and response mechanism according to the reaction with acetone gas; the sensing layer detects a signal that the electrical resistance (*R*) increases in proportion to the gas concentration along with the injection of acetone. The C8F molecule has repulsive properties against water molecules. The detected sensor's response and recovery times to acetone gas were simultaneously observed at room temperature to be 750 s and 500 s, respectively. The sensor's response and recovery time are defined as the time taken for the sensor to reach the 90% saturation response value and the time taken for recovery to 10%, respectively.

Acetone gas is found in trace amounts of less than 1 ppm in human exhaled gas, is linked to metabolism, and has been used as a medical biomarker for diabetes.^24–27^ Thus, it is a crucial requirement for tracing tens of ppb of acetone gas released during respiration. Humidity is present in both the ambient environment and exhalation, and the humidity dependence of respiratory gas sensing characteristics is a significant obstacle for real time applications. Thus, for practical applications including the point of care, the acetone sensor's high ppb level responsiveness and resistance to humidity remain critical tasks. Here, we manufactured an acetone gas sensor by drop-casting a C8F-doped PANi@HPMC CSS-NP ink solution onto a sensor substrate with a pair of gold electrodes (test cell: with a pair of integrated gold electrodes for a sensor electrode on a flexible substrate), as demonstrated in the sensor array diagram in Fig. 5. Particularly, C8F is placed on the surface of conductive PANi NPs as demonstrated in the figure and it not only works as a dopant but also has repulsive properties against water molecules due to its hydrophobic properties. Based on the study findings of Fujii *et al.*,^28^ it has been reported that C8F imparts hydrophobic properties when attached as a dopant on the surface of a conductive polymer including PPy. However, as demonstrated in the figure, the pure PANi@HPMC NP sensing material can detect the signal that the electrical resistance increases in proportion to the gas concentration with the C_3_H_6_O gas injection. Thus, with the adsorption of C_3_H_6_O gas, the charge density in the backbone of PANi decreases. At this time, C_3_H_6_O was thought to serve as a reducing agent to increase the resistance of PANi as a p-type semiconductor. The layer-structured films made of the as-prepared C8F-doped PANi@HPMC CSS-NPs demonstrate an electrical conductivity of 0.93 S cm^−1^ and largely enhanced humidity and temperature stability. At this time, the concentration of the C8F dopant was 4 × 10^−4^ wt% compared to PANi. However, the electrical resistance of PANi-based sensors influences the conductivity as the transfer of charged carriers within the PANi polymer chain and their resistance are changed by the adsorption of gas species.

When these chemical sensors are exposed to a certain gas, the sensor's resistance varies in proportion to the concentration of the adsorbed gas and measuring this is a fundamental principle. The PANi chain's electrical resistance increased as the amount of acetone increased. As demonstrated in Fig. 5, the sensing layer detected a signal indicating that the electrical resistance (*R*) increased in the positive direction along with the injection of acetone. However, the detection impact tended to decrease proportionally as the C8F concentration increased, and the detected sensor's response and recovery time to acetone gas were simultaneously observed to be 750 s and 500 s, respectively. The sensor's response and recovery time are defined as the time taken for the sensor to reach the 90% saturation response value and the time taken for recovery to 10%, respectively. At this time, while the response and recovery cycling tests were repeated up to 100 times, the change in sensitivity was observed to be less than 5%.

**Fig. 6:**
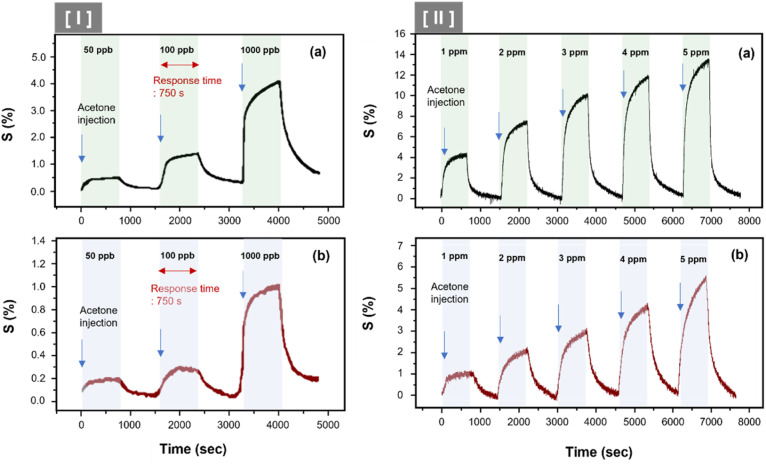
The continuous dynamic response of C8F-undoped-PANi@HPMC CSS-NP [(a) in the figure] and C8F-doped-PANi@HPMC CSS-NP [(b) in the figure] sensors to various concentrations of C_3_H_6_O gas ranging from 0.05–5 ppm at 25 °C and 0% relative humidity (RH), respectively. (I) and (II) The response spectrum in the 50–1000 ppb and 1–5 ppm concentration ranges, respectively.

**Fig. 7:**
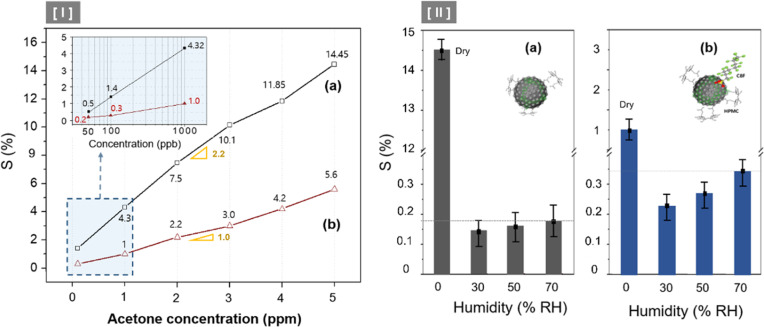
(I) Changes in sensitivity according to the concentration of acetone gas ranging from 0.05 to 5 ppm under dry conditions (0% RH); (a) C8F-undoped PANi@HPMC CSS-NPs and (b) C8F-doped PANi@HPMC CSS-NPs. (II) Changes in responsiveness (*S*%) at 2 ppm C_3_H_6_O concentration according to the changes under % RH (constant to *T* = 25 °C) conditions.

Fig. 6 illustrates the sensor array's response characteristics to C_3_H_6_O gas injection. We showed the continuous dynamic response of C8F-undoped-PANi@HPMC CSS-NP [(a) in Fig. 6] and C8F-doped-PANi@HPMC CSS-NP [(b) in Fig. 6] sensors to different concentrations of C_3_H_6_O gas ranging from 0.05 to 5 ppm at 25 °C and 0% RH, respectively. The response is characterized by a change in Δ*R*/*R*_i_ × 100 (responsiveness, *S*%) when injected with different concentrations of acetone, where *R*_i_ represents the initial current resistance, and Δ*R* represents the difference between *R*_i_ and *R* evaluated after exposure to acetone gas. As illustrated in the figure, the effective response was shown at each concentration (0.05, 0.1, 1, 2, 3, 4, and 5 ppm, respectively), and as the gas concentration increased, the relative responsiveness tended to increase in proportion. Particularly, as illustrated in Fig. 6(I), very prominent signals were observed even at trace gas concentrations of 50 ppb. In either case, the *S* was observed to have a linear characteristic toward increasing the resistance as the injection gas's concentration increased, as shown in Fig. 7(I). Then, the acetone gas sensor's sensitivity (ppm^−1^) can be defined as the slope of the response *S* against the concentration of acetone. The C8F-doped-PANi@HPMC CSS-NP sensor showed at least three times lower response to C_3_H_6_O gas compared to the C8F-undoped-PANi@HPMC CSS-NP sensor. This result can be explained by the fact that in the case of PANi doped with C8F, the reaction pathway with PANi was less activated since large-sized hydrophobic C8F molecules prevented C_3_H_6_O molecules' adsorption. Obtaining a reliable response to acetone at tens of ppb levels remains a crucial topic for biosensor applications that use exhalation.^29^

Almost all CCP-based sensors, including metal-oxide sensors, are easily influenced or do not respond under high-RH conditions. Thus, the sensing material's stability to the temperature and humidity environment functions as a major factor in evaluating the sensor's performance. In this sense, we observed the rate of changes in responsiveness (*S*%) at 2 ppm C_3_H_6_O concentration, according to the changes in RH% (constant to *T* = 25 °C) conditions, as illustrated in Fig. 7(II). In the case of the C8F-undoped PANi@HPMC CSS-NP sensor, as illustrated in Fig. 7(II-a), the *S* value under humidity conditions was significantly reduced to over one-twentieth of that under dry conditions. Furthermore, in the case of humidity conditions, the *S* value was stabilized at a low level or tended to increase slightly with the increase in humidity. Generally, PANi-based sensors have very high reactivity with water molecules, and when water comes into contact with the PANi backbone, the current resistance of PANI is substantially lowered. Thus, when several % of water molecules react in the C8F-undoped PANi@HPMC CSS-NP sensor, the sensor's current resistance (*R*) due to the reaction with acetone is offset and decreased due to their influence. However, in the case of the C8F-doped PANi@HPMC CSS-NP sensor (Fig. 7(II-b)), the *S* value under humidity conditions showed a relatively small decrease of 25–30% compared with dry conditions. Additionally, it demonstrated a tendency to increase proportionally with an increase in humidity. However, the resistance of the C8F-doped PANi@HPMC CSS-NP sensor was relatively less affected by humidity.

Furthermore, the *S* value slightly increases as the humidity increases because, under high-RH conditions, H_2_O molecules form small water droplets by self-binding, and these water droplets act to weaken the reactivity with the PANI backbone. According to previous reports,^31,32^ when gaseous C_3_H_6_O molecules come into contact with the small water droplet, there is a strong attractive force between the oxygen atom of C_3_H_6_O and the hydrogen atom of the water's OH group, so that acetone gas can be easily absorbed by the water droplet. This was also explained to be more affected by low-temperature conditions.

**Fig. 8:**
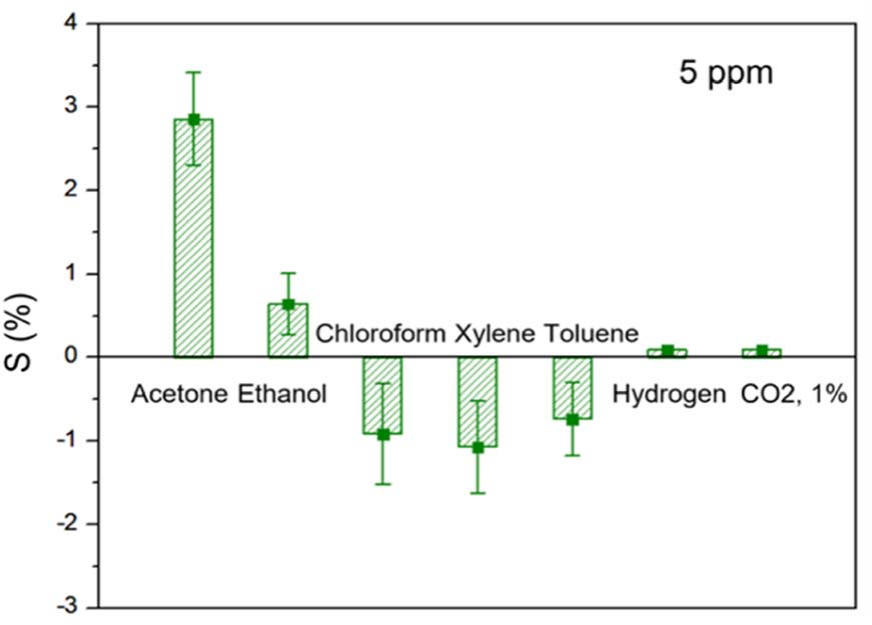
Comparison of response to several analytes at 5 ppm (acetone, ethanol, chloroform, xylene, toluene, hydrogen, and carbon dioxide).

Selectivity is another crucial parameter of gas sensors used in practice. To observe selectivity, our study further tested various organic gases such as ethanol, chloroform, xylene, toluene, hydrogen, and carbon dioxide. The concentrations of all gases employed for observation were 5 ppm (1% in CO_2_) and were measured at room temperature and under dry conditions. As illustrated in the comparative plot in Fig. 8, the C8F-doped PANi@HPMC CSS-NP sensor showed superior responsiveness (*S* = 2.8 at 5 ppm) for acetone, indicating very low responses. Furthermore, highly polar molecules such as acetone, ethanol, hydrogen, and carbon dioxide showed response characteristics that increased the electrical resistance of the PANi backbone. However, gases such as chloroform, xylene, and toluene showed negative responsiveness due to a decrease in electrical resistance in molecules with low polarity. Thus, in the case of the PANi-based sensor in this study, the change in electrical resistance was detected differently depending on the polarity of the gas component in contact. That is, highly polar molecules such as acetone, ethanol, hydrogen, and carbon dioxide show a response characteristic that increases the PANi backbone's electrical resistance. However, gases such as chloroform, xylene, and toluene show negative responsiveness due to a decrease in electrical resistance in molecules with low polarity.

Fig. 5 and 6 show the sensor's response and recovery curve. The reversibility of the response characteristics was also confirmed. When exposed to acetone at a concentration of less than 1 ppm, the sensor response was stable, and after that, a stable signal was confirmed even after 100 cycles of testing. This study's results show that the sensor's characteristics such as sensitivity, selectivity, humidity stability, repeatability and room temperature sensing are notable features. This work could yield good results of the comparative advantage compared with other PANi-based sensors reported in the literature,^13–15,20,30^ which sense at room temperature. As shown in the comparative data in Table 1, it was evaluated that the sensitivity (ppm^−1^) and detection limit (ppm) were very advanced.

**Table 1:** Comparison of this study with various PANi-based acetone gas sensors reported in the literature

Sensing materials	Sensitivity (ppm^−1^)	Detection limit (ppm)	Operating temp. (°C)	Type	Ref
Core-shell-shaped PANi@HPMC NPs	4.2	0.05	RT	P-type	This work
Core-shell-shaped C8F-doped PANi@HPMC NPs	1.0	0.05	RT	P-type	This work
PANi/Au/Al_2_O_3_	0.055	30	RT	P-type	20
PANi/TiO_2_	0.0035	20	RT	N-type	30
PANi fabrics	0.55	—	RT	P-type	13
PANi/Au/porous electrode	0.0039	23	RT	N-type	14
ZnQ/S–N: graphite QD/PANi	4	0.5	RT	N-type	15

## Experimental

### Synthesis of C8F-doped PANi@HPMC core–shell nanoparticles

With reference to a previous study, C8F-doped PANi@HPMC core–shell nanoparticles, as sensing materials, were synthesized.^19^ First, 5 ml of 0.169 M hydroxypropyl methylcellulose (HPMC, Sigma Aldrich) was dissolved in 5 ml of deionized (DI) water to prepare an aqueous dispersion, and then 100 μl of aniline monomer (GR, Sigma Aldrich) was added, followed by stirring at room temperature for 30 min. Second, 1 ml of 1 wt% HCl was added to the solution prepared above and stirred for 30 min, and 1.25 ml of 0.8 M ammonium persulfate (APS, 98.0%, Sigma Aldrich) was continuously added. Third, the reaction was conducted for 12 h without agitation at 0 °C. The reaction product is separated and purified by centrifugation at 15 000 rpm, and the obtained spherical nanoparticles are re-dispersed in DI water. Finally, 1 wt% C8F (40% in water solution, Sigma Aldrich) compared with aniline monomer was added to the dispersion, and the mixture was stirred at 300 rpm for 12 h or more. The synthesized core–shell-structured nanoparticle sensing materials were additionally washed two to three times with methanol and DI water and finally prepared as an ink solution of DI water dispersion.

### Characterization and analysis of acetone gas sensors

First, the as-prepared ink solutions of C8F-doped PANi@HPMC core–shell nanoparticles were directly coated onto a home-made test substrate (test electrode cell: silicone substrate with a pair of gold electrodes; the distance between the electrodes was kept at 0.5 cm) by adding dropwise and then drying at 80 °C in a vacuum oven for 10 min. Second, the prepared test sensor was placed in a gas chamber. To maintain dry conditions free of impurities, the chamber was purged for 20 min with pure nitrogen (N_2_) gas before testing. Finally, under varying concentrations (0.05–5 ppm), acetone gas mixed with dry air was injected. By setting a constant current in the sensor electrode, the resistance of the current flowing through the electrode was computed according to the DC-applied voltage response under different sensing conditions. The current resistance was measured in real time using a digital multimeter (Keithley 2000) by employing a DC voltage of 2 V under a temperature of 0–90% RH and 25–75 °C, respectively. The microstructure and detection mechanisms of C8F-doped PANi@HPMC core–shell nanoparticles were examined using SEM (JSM-633F, JEOL), TEM (JEN-1210, JEOL), and FT-IR (Nicolet 5700).

## Conclusion

In this study, we designed and synthesized a chemi-resistant acetone gas sensing material based on C8F-doped PANi@HPMC CSS-NPs with a core–shell shape and tested it at gas concentrations down to ppm. The C8F-doped PANi@HPMC CSS-NP sensors exhibited responsiveness to 50 ppb of acetone under ambient conditions, which could meet the clinical requirements for respiratory analysis. The sensing material examined using SEM, TEM, and several spectroscopic analyses reflected the novel sensing material's structural features as a layer of HPMC surrounding the PANi nanoparticles, and C8F on the outer surface of PANi imparted a specific water molecule repulsion effect. Particularly, the response to acetone gas was found to be good compared with other organic gas analytes. Furthermore, the detection stability was increased under high temperature and humidity conditions, which significantly affected the acetone reaction site. Finally, the C8F-doped PANi@HPMC CSS-NP's reaction mechanism with acetone gas was examined using UV/vis absorption and infrared spectroscopy.

## Conflicts of interest

The authors declare that they do not have any known competing financial interests or personal relationships that could influence the work reported in this paper.

## Supplementary Material
